# Stabilisation of the Fc Fragment of Human IgG1 by Engineered Intradomain Disulfide Bonds

**DOI:** 10.1371/journal.pone.0030083

**Published:** 2012-01-17

**Authors:** Gordana Wozniak-Knopp, Johannes Stadlmann, Florian Rüker

**Affiliations:** 1 Department of Biotechnology, Christian Doppler Laboratory for Antibody Engineering, University of Natural Resources and Life Sciences, Vienna, Austria; 2 Department of Chemistry, Christian Doppler Laboratory for Antibody Engineering, University of Natural Resources and Life Sciences, Vienna, Austria; Technical University of Braunschweig, Germany

## Abstract

We report the stabilization of the human IgG1 Fc fragment by engineered intradomain disulfide bonds. One of these bonds, which connects the N-terminus of the CH3 domain with the F-strand, led to an increase of the melting temperature of this domain by 10°C as compared to the CH3 domain in the context of the wild-type Fc region. Another engineered disulfide bond, which connects the BC loop of the CH3 domain with the D-strand, resulted in an increase of *T_m_* of 5°C. Combined in one molecule, both intradomain disulfide bonds led to an increase of the *T_m_* of about 15°C. All of these mutations had no impact on the thermal stability of the CH2 domain. Importantly, the binding of neonatal Fc receptor was also not influenced by the mutations. Overall, the stabilized CH3 domains described in this report provide an excellent basic scaffold for the engineering of Fc fragments for antigen-binding or other desired additional or improved properties. Additionally, we have introduced the intradomain disulfide bonds into an IgG Fc fragment engineered in C-terminal loops of the CH3 domain for binding to Her2/neu, and observed an increase of the *T_m_* of the CH3 domain for 7.5°C for CysP4, 15.5°C for CysP2 and 19°C for the CysP2 and CysP4 disulfide bonds combined in one molecule.

## Introduction

Monoclonal antibodies with high affinity and specificity are now well established therapeutics and invaluable tools for biological research. Among them, the IgG class is the most abundant one. This heterotetrameric protein is composed of two light chains and two heavy chains. Using proteolytic digestion, the IgG antibody can be divided into 2 antigen binding fragments (Fabs) and the Fc fragment. The Fc part itself is composed of two CH2 domains and two CH3 domains that form a homodimeric region at the C-terminal end of the antibody molecule. These regions associate with each other with two disulfide bridges in the hinge region which is located between the CH1 and CH2 domains and by strong non-covalent interactions between the two CH3 domains [1]. Two complex N-linked oligosaccharides are attached to the antibody through an asparagine residue in the CH2 domain. The oligosaccharides mediate the contact between the two CH2 domains. The CH2 domains harbour the binding sites for all effector molecules such as the Fcγ receptors as well as complement factor C1q. At the interface between CH2 and CH3 there is the binding site for neonatal Fc receptor (FcRn), which mediates the long half-life of antibodies.

The structure of antibody constant domains is similar to that of variable domains, consisting of beta-strands connected by loops, some of which contain short alpha-helical stretches. The framework is mostly rigid and the loops are comparatively more flexible, as can be seen from the b-factors of various Fc crystal structures (e.g. 1HZH.PBD and 1OQO.PDB). In the CH3 domain, the loops at the C-terminal tip of the globular domain are the sites which are most remote from the effector molecule binding sites and hence are the most attractive sites for engineering additional functionalities into the Fc fragment.

Recently, we have shown that it is possible to engineer antigen binding sites into C-terminal loops connecting beta-strands of CH3 domains, and that Fc fragments containing such modified CH3 domains, so-called Fcabs, possess all attractive properties of a classical IgG, at a size of approximately only 50 kDa [2]. Additionally, the paratopes of these CH3-mediated binding sites are sterically distinct from the classical Fab-mediated binding sites and could therefore interact with novel epitopes that are inaccessible to conventional antibodies or antibody fragments.

The plasticity of the Fc fragment that allows it to serve as a scaffold and to retain all antibody properties while accommodating the mutations in the loop regions is based on the fact that an Fc fragment *per se* is a very stable protein, with the CH3 domain being even more stable than the CH2 domain. Additionally, the two domains are generally believed to be independent from each other in their folding and structure [3], [4]. However, mutations in the loops, which are introduced for the purpose of creating an antigen binding site in the CH3 domain, can lead to a certain loss of stability of the domain due to novel unfavourable intramolecular interactions. Mutations leading to an increased stability of the bovine CH3 domains have been described [5]. In their paper, the authors argue that “a stabilised scaffold platform would provide more room for compromise when mutating for functional purposes”. An engineered disulfide bridge in an isolated CH2 domain of human IgG has been described to increase the melting temperature of this domain by almost 20°C [6], however no data have been shown on the effects of this disulfide bridge when it is placed in the context of a complete Fc. A llama single-domain antibody with an artificial disulfide bond has shown a 10°C higher midpoint temperature of thermal unfolding than the wild-type version of the molecule [7].

The Fc proteins described in this report were recombinantly expressed in the yeast *Pichia pastoris.* Although yeast glycosylation has an impact on the structure of CH2 domains and the activation of effector ligands (reviewed in [8]), the X-ray crystallographic analysis of Fc in complex with FcRn has revealed an interaction site at the inter-CH2-CH3 domain region [9], [10] that is minimally affected by the glycosylation status [11]. It has been demonstrated that the interaction of Fc with immobilized FcRn can best be modelled as Fc binding to two classes of non-interactive sites on FcRn, and that the observed affinities are higher when FcRn, rather than Fc, is immobilized [12], [13]. In our present report we resorted to the above experimental outlay to monitor pH-dependent binding of Fc fragment to FcRn.

We have succeeded to increase the stability of the CH3 domain of human IgG1 by the introduction of additional intradomain disulfide bonds. Results on the thermal stability as well as neonatal Fc receptor binding properties of Fc fragments carrying single as well as combined engineered intradomain bonds are presented in this report. To show that thermal stabilization with the described disulfide bonds is also applicable to an Fc fragment with a newly introduced antigen binding site, we describe the effect of each novel disulfide bond as well as the combination of both in the model Fcab H10-03-6 which binds specifically to Her2/neu [2]. The antigen binding site has been engineered into this Fc fragment by modifications in the AB and EF loops of the CH domain, and has led to a decrease of the thermostability of this protein. We show in this report that the engineered disulfide bonds work not only in wild-type Fc but were also successful in reestablishing the thermostability of Fcab H10-03-6.

## Results

### Design of engineered human Fc fragments with additional disulfide bonds in the CH3 domain

We aimed at increasing the stability of the human CH3 domain in the context of an Fc fragment, in order to increase its tolerance to mutations in loop regions, which may be introduced at a later stage with the purpose of creating antigen binding sites in this unconventional region of the antibody molecule. For this purpose intradomain disulfide bridges were designed.

Out of 29 disulfide bonds that were predicted by DSDBASE to be possible in the CH3 domain, we selected 5 that seemed to be the ones with the highest likelihood of success as judged by visual examination of the crystal structure of an Fc fragment: Thr350 and Leu441 (CysP1), Pro343 and Ala431 (CysP2), Ser375 and Phe404 (CysP3), Ser375 and Pro396 (CysP4) and Val348 and Lys439 (CysP5). All residue numberings in this paper are according to Kabat [14].Three of these constructs either did not show increased thermostability (CysP1, CysP5) or led to aggregation of the recombinant protein (CysP3), and were consequently not analyzed further,. However 2 of the selected mutants, CysP2 and CysP4, displayed a significantly higher thermostability than wild-type Fc and furthermore showed a wild-type like profile in size exclusion chromatography (HPLC), indicating a stabilized fold and the absence of aggregation.

**Figure 1:**
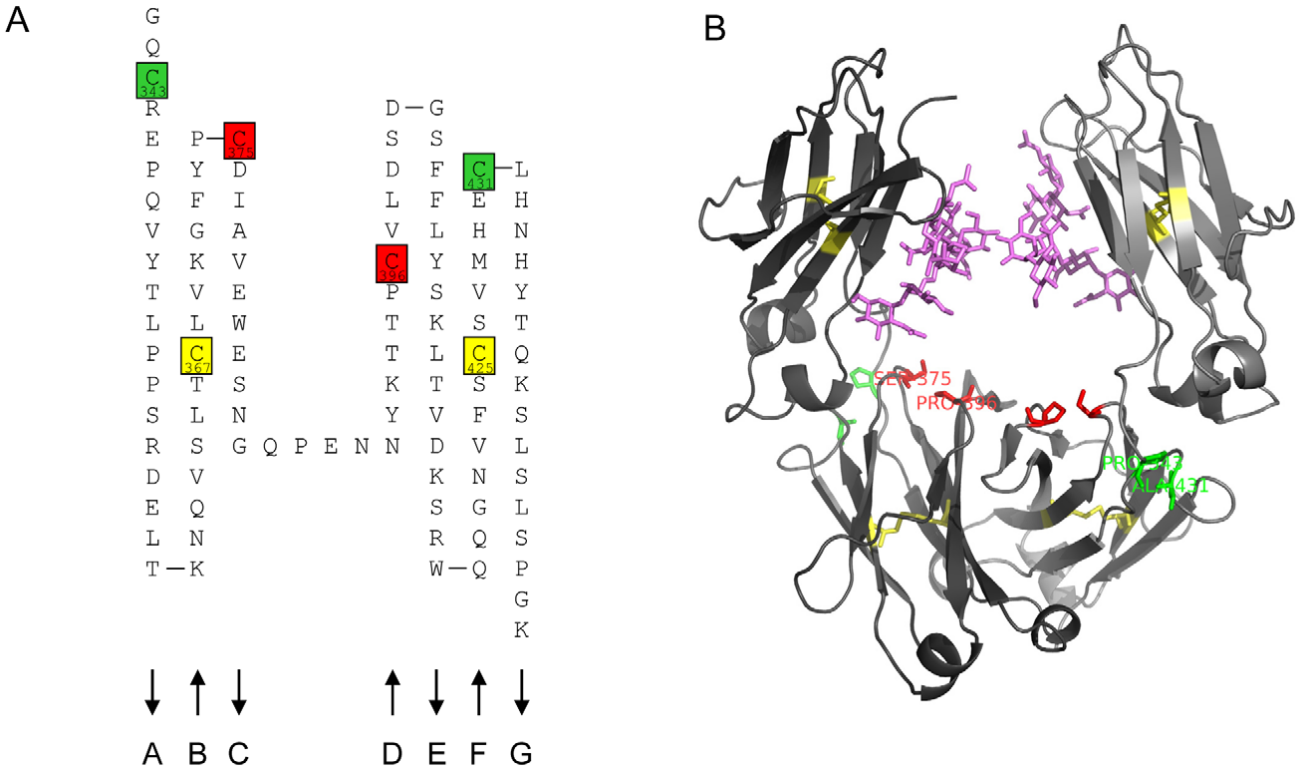
Graphical presentation of secondary and tertiary structure of the CH3 domain and of an Fc fragment, and location of engineered disulfide bonds. A: 2D-presentation (“Collier de perles” [32]) of the secondary structure of the CH3 domain. The residues that were mutated to Cysteine to form the engineered disulfide bonds are indicated in green (CysP2) and red (CysP4). Native disulfide bond residues are indicated in yellow. B: 3D cartoon presentation (created using the PyMOL Molecular Graphics System, Version 1.3, Schrödinger, LLC) of the human IgG1 Fc fragment (Protein Data Bank (http://www.pdb.org/) accession 1OQO) with the positions that were mutated to create the engineered disulfide bonds indicated in green and red as in Figure 1A. The native disulfide bonds are shown in yellow.

In CysP2, the N-terminus of the A strand and the C-terminus of the F strand are disulfide-bonded while in CysP4 the engineered disulfide bond connects the BC loop and the D-strand. The location of the stabilizing disulfide bridges is shown in a 2D presentation of the secondary structure of the CH3 domain in Figure 1A and in a 3D cartoon presentation of the Fc fragment in Figure 1B.

### Expression and characterization of human Fc expressed in *P. pastoris*

The gene coding for human Fc was cloned in the *P. pastoris* expression vector pPICZalphaA, expressed and purified by Protein A affinity chromatography as described in Materials and Methods. All the mutants described here readily expressed at high levels as soluble proteins, similar to wild-type Fc, indicating their overall fitness. They were soluble in aqueous buffer at a concentration of up to 5 mg/ml similar to wild-type Fc and had a dimeric conformation as determined by size exclusion chromatography under native conditions. On SDS-PAGE (see Figure S1), the proteins run at about 55 kDa, indicating the presence of N-linked glycosylation at residue Asn297 as expected.

### Thermostability of wild-type Fc, CysP2, CysP4 and CysP24

The thermal denaturation of human IgG1-Fc in PBS, pH 7.4 over the temperature range 5–100°C is characterised by two endothermic transitions, at 65°C and 81.9°C. The first transition has been described to be reversible as shown by heating up to 75°C, cooling and again heating to 90°C, whereas the second transition is irreversible. However, beyond 75°C, the CH2 transition also becomes irreversible [4].

**Figure 2:**
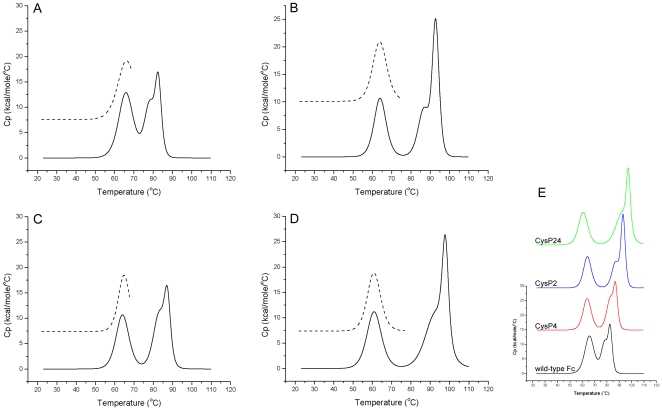
Thermograms of wild-type and disulfide bond stabilized Fc mutants obtained with DSC measurements. A: wild-type Fc, B: FcCysP2, C: FcCysP4, D: FcCysP24, E: overlay of all 5 proteins. For each protein the point of reversibility of melting of CH2 domain was determined which is shown as a dashed line in the inset graphs.

**Figure 3:**
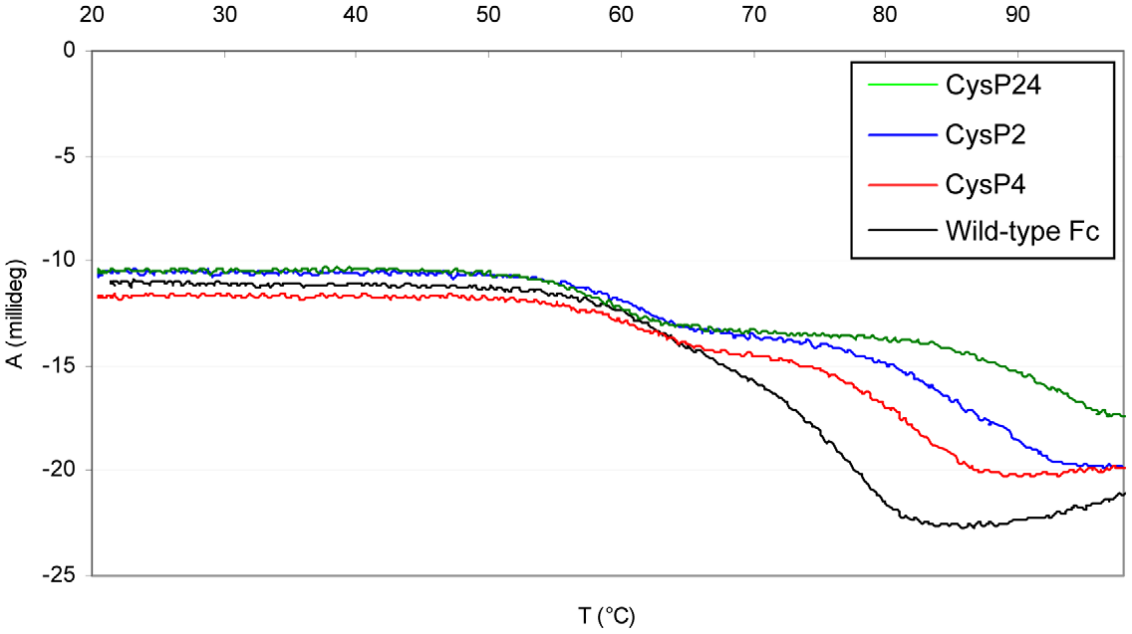
Thermal unfolding curves of disulfide bond stabilized Fc-mutants obtained from tCD-measurements. Experiments were carried out at a protein concentration of 570 µg/ml in PBS at 218 nm using a 1-mm cell.

To test the thermal stability of Fc fragments expressed in *P. pastoris*, both Differential Scanning Calorimetry (DSC) and temperature-dependent Circular Dichroism (tCD) were used. The results of these measurements are shown in Figure 2 (DSC) and Figure 3 (tCD), the thermodynamic parameters of melting of wild-type and mutated Fc fragments derived from DSC data are given in Table 1, and the melting temperatures derived from tCD measurements are given in Table 2. By DSC, the *T_m_* of the CH2 transition of wild-type Fc was at 65°C, which is 4°C lower than that of Fc expressed in mammalian cells. This can be attributed to differences in the glycosylation pattern between the two expression systems. The thermal transition of the wild-type CH3 domain could be fitted with two non-2-state transitions at 77.8°C and 82.6°C, the first event corresponding likely to the melting of CH3 dimer and the second transition representing the denaturation of CH3 domains. As measured by CD, the secondary structure of the Fc fragment at 25°C is populated primarily of beta-strands; therefore thermal denaturation was followed by tCD at 218 nm. Unfolding started at 54°C and was completed at 82.5°C. A double sigmoid model was used to obtain *T_m_* values at 65.2°C and 77.0°C.

**Table 1:** Transition temperatures of melting of wild-type Fc and Fc fragments with novel disulfide bonds derived from DSC data.

	Temperature (°C) of transition			Point of reversibility of CH2 unfolding (°C)
	1^st^	2^nd^	3^rd^	
wild-type Fc	65.9	78.1	82.6	68.5
CysP2	64.0	86.6	92.8	73.0
CysP4	64.1	82.9	87.3	67.5
CysP24	61.0	92.8	97.8	75.0

**Table 2:** Melting temperatures of wild-type Fc and Fc fragments with novel disulfide bonds derived from tCD data.

	Temperature (°C) of transition	
	1^st^	2^nd^
wild-type Fc	65.2	77.0
CysP2	61.0	85.1
CysP4	62.1	80.7
CysP24	58.8	90.4

In all cases, the three mutants CysP2, CysP4 and CysP24 were remarkably more stable than wild-type Fc. Notably, 50% unfolding of the CH3 domains of CysP2, CysP4 and CysP24 mutants occurred at temperatures that were significantly higher than that of wild-type Fc. In the case of CysP4, the second and the third transition appeared at temperatures 5°C higher than with wild-type Fc. The stabilization effect was even more pronounced in the case of CysP2, where the second and the third transition appeared at 9°C higher temperature than with wild-type Fc. The DSC profile of the Fc fragment CysP24, in which the CysP2 and CysP4 mutations are combined exhibited the second transition at 92.8°C and the third transition at 97.8°C, which is 15°C above the ones characteristic for wild-type Fc. Interestingly, in all cases thermal unfolding of the CH2 domain was reversible up to the temperature point where melting of the CH3 domain started, which was probed by annealing scans (Fig. 2 A–D and Table 1). In both mutants with additional single intradomain cysteine bridges, the *T_m_* of the CH2 domain was slightly decreased by 1.5°C, and in the double mutant it was decreased by 4°C.

Far-UV CD-spectra of wild-type Fc, CysP2, CysP4 and CysP24 were identical at 25°C, therefore a wavelength of 218 nm was chosen for all proteins to evaluate thermal unfolding by CD measurement. Thermal denaturation curves could be fitted with a double sigmoid model to obtain the *T_m_* values, which have shown the same trend in stabilization as the data obtained from DSC measurements (Table 2).

### Mass spectrometric analysis

In order to show the presence of the correct disulfide bonds, as well as the absence of incorrect ones, mass spectrometric analysis was performed with samples CysP2 and CysP4. From the comparative analysis of the reduced and the non-reduced peptide-sample, a number of disulfide-bonded di-peptide masses were identified. Based on the MS/MS data of the respective reduced peptide masses, the amino-acid sequences of these peptides were determined. Subsequently, the theoretical masses of all possible combinations for the consequential hypothetical cysteine-linked di-peptides were calculated using the following formula:
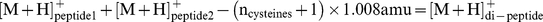
The XICs of the hypothetical masses allowed the identification of the di-peptides which were actually present in the samples.

Both samples, CysP2 and CysP4, exhibited doubly and triply charged ions of the mass 2602.19 amu, which corresponds to the homo-di-peptide EFTC*PPC*PAPEL, linked by two disulfide bonds, which is derived from the hinge region of the Fc (see Figure S2A for CysP2 and Figure S2B for CysP4 data). Furthermore, in both samples doubly, triply and fourfold charged ions of the mass 3075.66 amu were found, which reflects the di-peptide resulting from the disulfide bond between the peptide ISRTPEVTC*VVVD (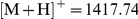) and YKC*KVSNKALPAPIE (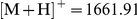), which corresponds to the native disulfide bond in the CH2 domain (see Figure S2A).

In contrast to CysP2, where neither of these ions were detected, CysP4 yielded ion masses confirming the disulfide bonds between the peptides derived from the engineered disulfide bond in CysP4, VKGFYPC*D (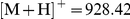) and YKTTPC*VL (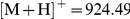). Furthermore, the peptides derived from the native disulfide bond in the CH3 domain, FSC*SVMHEALHNHYTQKSLSLSPGK (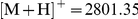) and TC*L (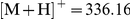) were detected. In turn, however, the masses corresponding to the engineered disulfide bond between the peptide KTISKAKGQC*REPQV (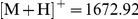) and HEC*LHNHYTQKSLSLSPGK (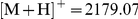) were exclusively detected in the sample CysP2. However, no ions confirming the native CH3 disulfide bond in CysP2 disulfide bond could be detected, which can be interpreted as follows: the native disulfide bond within the CH3 domain of CysP4 was found to span between the C-terminal peptide of the amino-acid sequence FSC*SVMHEALHNHYTQKSLSLSPGK and the peptide TC*L. The corresponding native disulfide bond within CysC2 would thus be expected to also link the C-terminal peptide of CysP2, namely FSC*SVMHEC*LHNHYTQKSLSLSPGK, to the peptide TC*L. However, due to the presence of the engineered, second cysteine residue within this CysP2 derived C-terminal peptide, which was found to be also linked to the peptide KTISKAKGQC*REPQV, our experimental conditions should yield a fairly unconventional analyte comprising three S-S-interlinked peptides (of approx. 4800.3 Da). We were not able to confidently detect this structure in the course of the present study. Alternatively, as indicated in Figure S2, pepsin also generates a shorter C-terminal peptide variant of CysP2, namely HEC*LHNHYTQKSLSLSPGK, which was clearly found to be linked to the peptide KTISKAKGQC*REPQV. This suggests that the native disulfide bond of the CH3-domain would also give rise to an S-S link between the two peptic peptides FSC*SVM and TC*L. These two latter peptides were not detected in our analysis, which can be explained by the fact that they are rather short and hydrophilic, and might thus fail to be retained on a conventional C18 column. However, despite the lack of direct mass-spectrometric evidence for the formation of the native CH3 S-S in CysP2, we can confidently assume its native structure because all other cysteines within the molecule have been assigned to discrete disulfide-bonds (i.e. applying Ockham's razor or lex parsimoniae) and, furthermore, unliganded, free cysteine residues within the protein would lead to misfolding, multimerization or aggregation. As shown below, neither of this was observed in CD spectral analysis, or size-exclusion chromatography.

### Structural characterisation of CysP2, CysP4 and CysP24

**Figure 4:**
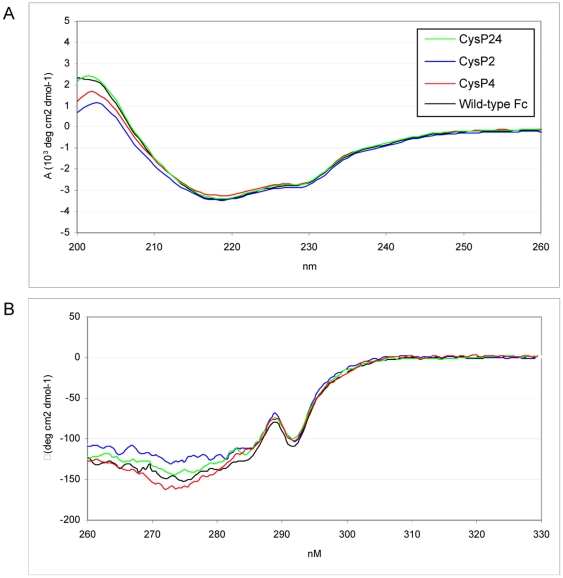
Spectroscopic characterization of disulfide bond stabilized Fc-mutants: (A) far-UV CD spectra and (B) near-UV CD spectra of stabilized Fc-mutants. The measurements were carried out at a protein concentration of 570 µg/ml in PBS at 25°C.

To investigate the effect of the disulfide bonds on the conformation of the Fc fragments, far- and near- UV CD spectra were obtained for all mutants. The results are shown in Figure 4A and 4B respectively. It can be seen that the Fc fragments with additional disulfide bonds have intact secondary and tertiary structures that are very similar to the wild-type Fc fragment, with a characteristic minimum at 218 nm. The far CD spectrum shows a weak and broad minimum at about 223 nm, a band that is traditionally attributed to the absorption of alpha helix. However in a protein with a low α-helix content such as Fc, changes in this band are indicative of changes in the conformations of aromatic side-chains [15].

In the near CD-spectrum, the main minimum was at 273 nm and a second minimum was observed at 293 nm. Near-UV spectra were similar for all proteins and had similar absolute intensities, suggesting that the tertiary structures of Fc and its disulfide-stabilized variants described here may not be significantly different.

**Figure 5:**
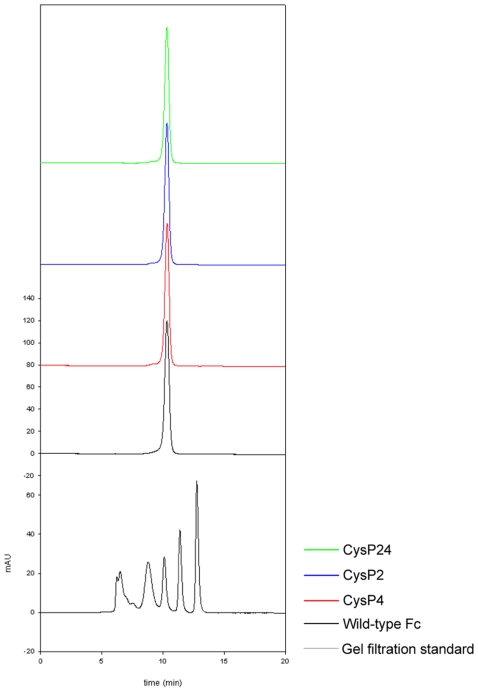
Size exclusion chromatograms of wild-type Fc and disulfide bond stabilized Fc-mutants monitored at A_280_.

By size exclusion chromatography (Figure 5), we have established that the disulfide-stabilized Fc variants CysP2, CysP4 and CysP24 have identical elution profiles as wild-type Fc. The result was further corroborated by using various molecular weight standards. This observation implies that the introduction of novel disulfide bonds in the human CH3 domain leads to stabilized Fc variants that have very similar overall structural parameters as compared to wild-type Fc. The dimer interface is intact and no signs of aggregation are observed, which could potentially be caused by mispairing of cysteine residues.

### Binding of FcRn to the stabilized Fc mutants

**Figure 6:**
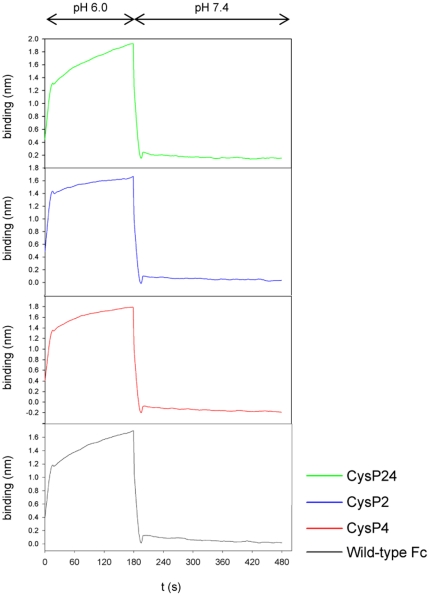
BLI analyses of FcRn interaction with wild-type Fc and with disulfide bond stabilized Fc-mutants. Biotinylated FcRn was immobilized to streptavidin tips. Association of the Fc fragment was observed at pH 6.0 and a sharp dissociation was induced by shifting the pH to 7.4.

To examine an eventual impact which the newly introduced cysteine mutations could have on the binding of the Fc variants to the neonatal Fc receptor (FcRn), this interaction was studied using Biolayer Interferometry (BLI, ForteBio Octet). Association to FcRn, the binding site of which maps to the interface between the CH2 and CH3 domains, was assessed at pH 6.0, whereas the biologically important dissociation was measured by shifting the pH to 7.4. None of the mutants show any observable difference to wild-type Fc (Figure 6).

### Stabilization of Her2/neu binding Fcab H10-03-6 with CysP2 and CysP4

**Figure 7:**
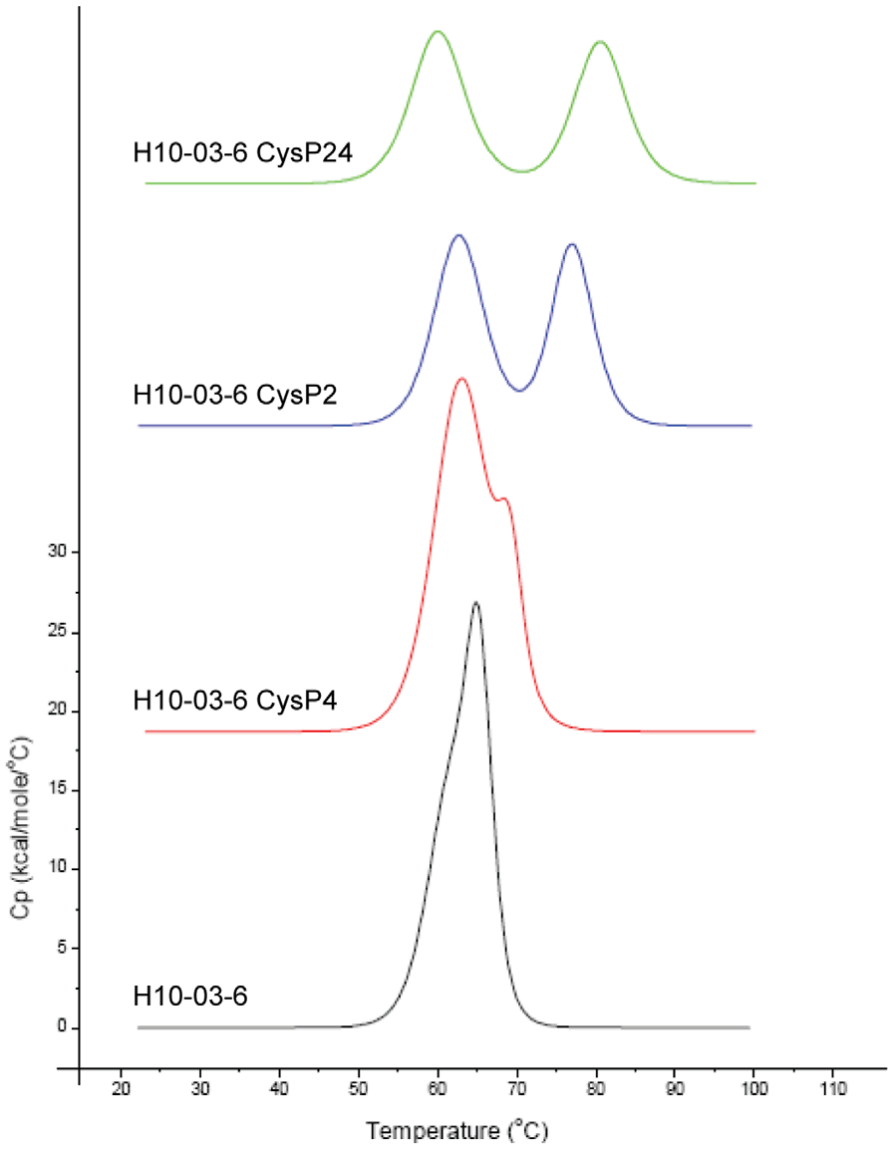
Thermograms of Her2/neu binding Fcab H10-03-6 and its disulfide bond stabilized derivatives obtained with DSC measurements.

For the Fc fragment H10-03-6, which binds to Her2 with about 70 nM affinity, a decrease in thermal stability comparing to wild-type Fc has been observed. In this case irreversible thermal unfolding can be deconvoluted with two transitions, corresponding to melting of the CH3 domain at 61.6°C and of the CH2 domain at 65.3°C. Each of the three mutants H10-03-6-CysP2, -CysP4 and -CysP24 was more stable than the parent clone. In the case of H10-03-6-CysP4, the transitions were still overlapping and by deconvolution appeared at 62.9°c and 69.0°C. The stabilization in the case of H10-03-6-CysP2 was more effective as the transitions appeared at 62.7°C and 77.0°C. The mutant with combined novel disulfide bridges H10-03-6-CysP24 has shown transitions at 60.0°C and 80.3°C, which is 19°C above the Tm of the CH3 domain of the unmodified H10-03-6 CH3 domain (Figure 7).

## Discussion

A wide variety of strategies and methods has been successfully applied to increase the thermal stability of proteins, including random mutagenesis combined with *in vitro* directed evolution [16], rational or computational design [17], [18], or statistical analysis [5], [19]. Engineered disulfide bonds have been used for stabilization of several globular proteins [20], and in particular for antibody fragments such as variable domains [7], [21]. In one example, it was shown that the introduction of an additional disulfide bond between strands C′ and D, and C and E, naturally occurring or engineered in the llama heavy chain variable domains can significantly increase their stability [22]. In another example, also a constant domain was stabilized [6]. In this latter publication, a remarkable positive shift in the *T_m_* of a human isolated IgG1 CH2 domain was described. However, it remains to be demonstrated that this stabilized CH2 domain is also suitable as a module in an Fc or in a complete immunoglobulin.

We have recently shown [2] that antigen-binding sites can be introduced in C-terminal loops of the CH3 domain of human IgG1. Experience from selections of antigen-binding Fc fragments has indicated to us that some of the mutations that may be introduced in loop regions with the purpose of creating an antigen binding site can lead to a decrease of thermostability and of the folding properties of the Fc fragment. With this in mind, we attempted to develop a generic approach to increase the intrinsic stability of the CH3 domain in the context of Fc by introduction of stabilizing intradomain disulfide bridges.

The introduction of the additional disulfide bonds described in this report has led to a significant increase in thermostability of the Fc, while leaving all other measured properties unchanged. Expression characteristics were identical for all proteins studied, and structural parameters as assessed by size exclusion chromatography as well as far- and near-UV CD measurements were indistinguishable. Binding to neonatal Fc receptor also remained unchanged. A slight decrease in the temperature of the first transition (1.5°C for CysP2 and CysP4, and 4°C for CysP24) was however observed. It has been proposed that the intensity of the first heat absorption peak is dependent on melting of the CH2 domain polypeptide, interaction of the CH2 domains and of the cis interaction of CH2 and CH3 domains [23]. As all mutated proteins have shown identical folding, we cannot attribute lowering of the T_m_ to an unfolded CH2 domain. Stabilization of CH3 dimer, for example due to “compacting” at low pH values, however can weaken the cis interactions [3]. This could be the likely cause for the decrease in temperature of the first transition in the case of Fc fragments that have been stabilized by disulfide bonds in the CH3 domain.

The binding of FcRn was used as an indicator of correct folding of the Fc and of the retention of its remarkably long *in vivo* half-life. For example, it has been shown for a *Pichia* expressed scFv-Fc fusion protein that its half-life was extended to 93 h in mice in comparison to 3.5 h for a typical stand-alone scFv [24]. No change in the association process for the ligand in comparison to wild-type Fc was observed, and binding of FcRn at pH 6.0 and its pH-dependent release at pH 7.4 was also unaffected. Yeasts of the genus *Pichia* in general attach high-mannose structures, most commonly Man_14_GlcNAc_2_ as compared with even longer (over 50 mannose residues) chains in *S. cerevisiae* proteins [25]. Modified carbohydrates can influence protein structure primarily in the proximity of the N-terminal region of the CH2 domain, which may imply altered reactivity to effector molecules. In contrast, FcRn which binds mainly to the inter-CH2-CH3 domain region, in a ratio of one FcRn dimer to one Fc molecule [9], [10] is known to be largely unaffected by the glycosylation status of the Fc [11].

CysP2 (delta*T_m_* 10°C), in which the N-terminus of the A strand and the C-terminus of the F strand are disulfide-bonded showed a higher degree of stabilization in the CH3 domain than CysP4 (delta*T_m_* 5°C), in which the BC loop and the D-sheet were connected. The contribution of disulfide bonds to protein stability is presumed to be mainly due to loss of conformational entropy in the unfolded state [26], assuming the enthalpy change is negligible. According to Pace [27], the entropic loss by a disulfide bond of an unfolded protein can be estimated using the following equation for the conformational entropy deltaS:
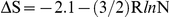
where N is the number of residues in the loop forming the disulfide bond. When the naturally occurring disulfide bond in the CH3 domain was reduced experimentally, good agreement between the theoretical TdeltaS value and the experimentally determined deltaG difference between reduced and oxidized forms (−4.1 kcal/mol versus −3 kcal/mol) was found [28]. It was therefore deduced that the majority of the stabilization energy provided by the disulfide solely stems from its contribution to the unfolded state, assuming that the difference in the free energy change between the two forms in the folded state is small.

Although these computations cannot be applied bona fide to the Fc fragment as described here, due to the irreversibility of the unfolding of the CH3 domain, it may give a hint as to why a considerable difference in the degree of stabilization between the two intradomain disulfide bond constructs is observed: in the case of CysP4, there are only 20 residues between the two cysteine residues which form the bond, hence deltaS at 20°C (TdeltaS) is estimated to be −3.2 kcal/mol whereas in the case of CysP2, there are 85 residues in the loop forming the disulfide bond, yielding an estimate of −4.5 kcal/mol.

Another interesting observation is that with the increase in *T_m_* of the denaturation of the CH3 domains, also the *T_m_* of the second CH3-associated transition has increased. The most common explanation for this transition is the event of dimer dissociation. We therefore conclude that the newly introduced disulfide bridges not only stabilized the conformation of the monomeric CH3 domain but also enhanced the CH3/CH3 homodimer interaction. This is in accordance with reports that have shown that native disulfide bond formation in human CH3 domains is critical for both, stability and dimerization [28], and this data is also supported by other reports [29], [30].

The results presented in this paper have implications for the development of stabilized Fc fragments which can be used as stand-alone scaffolds or as parts of whole antibodies. Mutations are routinely introduced in antibodies in order to enhance their effector functions or to achieve longer *in vivo* half-life. Such mutations may cause destabilizing effects due to slight alterations in the interaction network of chemical bonds within the protein domain. Stabilization efforts such as the ones described here can counteract such unwanted effects and can therefore allow a higher degree of freedom for mutagenesis.

All stabilizing mutations described here resulted in stabilized Fc fragments with wild-type like expression properties, displaying homodimeric proteins without tendency for aggregation, which is an important prerequisite for potentially therapeutic proteins. Importantly, the engineered disulfide bridges also exerted their stabilizing effects on a model antigen-binding Fcab selected in our lab. Interestingly, it was possible also in this case to combine the stabilization effect of both novel disulfide bonds. Further studies will be performed to investigate whether higher thermal stability also correlates with greater stability *in vivo*.

## Materials and Methods

### Ethics statement

Under Austrian law and under the internal rules of the University of Natural Resources and Life Sciences, Vienna, the work described here did not require permission by an ethics committee. No animal experiments were performed.

The necessary declaration of work involving the use of genetically modified microorganisms was performed at the Committee for Biological Safety of the Department of Biotechnology, which subsequently did not forbid the described experiments (declaration number IAM 2007_03).

### Design of intradomain disulfide bridges

Positions in the CH3 domain sequence that could be mutated to cysteine in order to create disulfide bridges were predicted using DSDBASE [31] (http://caps.ncbs.res.in/dsdbase/dsdbase.html). 1OQO.PDB was used as a structure of the Fc fragment of human IgG1. The DSDBASE algorithm identifies residue pairs with C_alpha_-C_alpha_ distances of less than or equal to 6.5 Å and C_beta_-C_beta_ distances of less than or equal to 4.5 Å and evaluates the proposed disulfide bridges using limits for the structural parameters of the various torsion angles and S-S bond length. For the monomeric CH3 domain, 29 potentially suitable positions were found based on these parameters. All proposed disulfide bridges were evaluated by visual inspection of the crystal structure of the Fc fragment (1OQO.PDB) and 5 (CysP1–CysP5) were subsequently chosen for experimental evaluation.

### Expression and purification of the human IgG1 Fc fragment

The Fc fragment of human IgG1 was cloned in the expression vector pPICZalphaA (Invitrogen) and transformed to the AOX locus of *Pichia pastoris* strain X33 after linearization with *Sac*I. The amino acid sequence of the human IgG1 Fc fragment is as follows:

EFTCPPCPAPELLGGPSVFLFPPKPKDTLMISRTPEVTCVVVDVSHEDPEVKFNWYVDGVEVHNAKTKPREEQYNSTYRVVSVLTVLHQDWLNGKEYKCKVSNKALPAPIEKTISKAKGQPREPQVYTLPPSRDELTKNQVSLTCLVKGFYPSDIAVEWESNGQPENNYKTTPPVLDSDGSFFLYSKLTVDKSRWQQGNVFSCSVMHEALHNHYTQKSLSLSPGK

The cloned sequence contained 2 extra amino acid residues (EF) at the N-terminal site due to the *Eco*RI site used for cloning. The threonine at position 3 corresponds to amino acid position 225 according to Kabat EU numbering.

The recombinant protein was expressed in *P. pastoris* X33 in the presence of 1% methanol and induction was continued up to 5 days. The supernatant was harvested by centrifugation, loaded onto a Protein A HP column (GE Healthcare), eluted with 0.1 M glycine, pH 3.5, and subsequently dialysed against PBS at 4°C. The protein concentration was determined by UV spectroscopy using an absorption coefficient at 280 nm of 1.397 for wild-type Fc dimer.

### Mutagenesis

Mutagenesis was performed using the QuikChange® Multi Site-Directed Mutagenesis Kit (Stratagene), according to the manufacturer's instructions. The primers used are listed in File S1.

### Proteolytic degradation of samples and differential peptide mapping by mass spectrometry

In order to prevent the potential scrambling reactions of disulfide bonds during the proteolytic degradation, the samples were digested at acidic pH. For this, 20 µL of the respective Fc sample (0.5 mg/mL) were adjusted to 1% formic acid final concentration, by the addition of 20 µL of 2% formic acid in water, containing 10 µg/mL pepsin (from porcine gastric mucosa, Sigma-Aldrich). Prior to the digestion at 37°C, over night, the resulting pH (i.e. approx. pH 2) was briefly checked on pH paper.

The peptic peptide maps were produced by LC-ESI-MS/MS, using a Dionex Ultimate 3000 LC-system, coupled to a Waters Q-TOF Ultima equipped with its standard ESI-source. 5 µL aliquots of the proteolytic digests were directly loaded onto a BioBasic C18 column (0.32×150 mm, 5 µ, Thermo) at a flow rate of 6 µL 60 mM ammonium formate, pH 3.0, per minute, and separated by developing a linear gradient from 0 to 80% AcCN (in 60 mM ammonium formate, pH 3.0), over 80 minutes, at a flow rate of 6 µL per minute.

For MS analysis of the peptic peptides, the capillary voltage was set to 3.2 kV, the cone voltage to 80 V, the source temperature to 100°C and the desolvation temperature to 120°C. Positively charged ions were recorded in the m/z range from 50 to 2000. The MS/MS experiments were performed in the data dependant acquisition mode, automatically selecting the respective ions, as well as CID energies. MS and MS/MS data were manually evaluated using the MassLynx 4.0 software.

In order to identify the elution time points of the disulfide-bonded di-peptides in the peptide maps based on their susceptibility to reducing conditions, 5 µL aliquots of the peptic digests were dried in a speed vac, and taken up in 5 µL 100 mM NH_4_HCO_3_, containing 10 mM DTE. The reduction was then allowed to stand for 30 minutes at 56°C, before the reduced samples were directly subjected to LC-ESI-MS/MS analysis.

### Differential scanning calorimetry

Differential Scanning Calorimetry experiments were performed using an automated VP-Capillary DSC Microcalorimeter (MicroCal, Northampton, MA), using 5 µM protein solutions, diluted in PBS at pH 7.4 The heating rate was 1°C/min, and the heating was performed from 20°C to 110°C. Fitting was performed using the non-2-state transition mechanism. The highest temperature that allowed thermal denaturation of the CH2 domain was first explored using annealing scans, with the first one from 20°C to the temperature point at which the start of melting of the CH3 domain was observed as indicated by the deconvolution of the thermogram, followed by cooling of the sample in situ, and a second scan from 20°C to 110°C. Data were confirmed by controls runs in which the highest permissible temperature was exceeded by 1.5°C.

### CD spectroscopy

The circular dichroism (CD) spectra were measured on a Chirascan spectropolarimeter (Applied Photophysics). A 1 mm path-length quartz cuvette was used for far-UV spectra measurement, and a 10-mm path-length quartz cuvette was used for the near-UV spectra measurement. The protein concentration was 570 µg/ml, and the buffer used was PBS. The CD spectra of the buffer solution were subtracted from the sample spectra before conversion to absolute CD values.

Thermal unfolding was monitored by measurement of temperature-dependent circular dichroism (tCD) on a Chirascan spectropolarimeter (Applied Photophysics). A 1 mm quartz cuvette was used at a wavelength of 218 nm. All measurements were performed form 25 to 98°C with 0.2°C increment at a heating rate of 1°C/min.

### Size-exclusion HPLC

A Shimadzu LC-20A Prominence system equipped with a diode array detector and a refractive index detector was used to perform size-exclusion HPLC with A TSK G3000 SWXL column (7.8×30 mm). The mobile-phase buffer used was PBS, 200 mM NaCl. Chromatography was conducted with a constant flow-rate of 1 ml/min. A total of 50 µg protein at about 1 mg/ml were loaded on the column for analysis.

### Biolayer Interferometry

ForteBio Octet was used for observation of the interaction of disulfide-stabilized Fc mutants with FcRn. Streptavidin-coated tips (ForteBio) were loaded with biotinylated FcRn at 5 µg/ml. Fc fragments at a concentration of 4.8 µM were allowed to associate in PBS, pH 6.0, and dissociation from FcRn was monitored in PBS, pH 7.4, at 30°C and with shaking at 1000 rpm. Binding of Fc fragments to an uncoated tip was used as a reference. Data were analysed with ForteBio Data Analysis package 6.2. *In vivo* biotinylated FcRn was refolded from bacterial inclusion bodies and was a kind gift of Sune Justesen (University of Copenhagen).

### Mutagenesis of Her2/neu binding Fcab H10-03-6

Mutagenesis was performed using the QuikChange® Multi Site-Directed Mutagenesis Kit (Stratagene), according to the manufacturer's instructions. The primers used are identical to the primers used for Fc wild-type, except for the mutation S375C, and are listed in File S1. Modified Fc fragments were cloned, expressed and purified and thermograms measured by DSC as described above for wild-type Fc fragments.

## Supporting Information

Figure S1 SDS-PAGE of purified wild-type Fc (lane 1), CysP4 (lane 2), CysP2 (lane 3) and CysP24 (lane 4). Molecular weight standard (lane M).
(TIF)

Figure S2 MS/MS data enabling prediction of disulfide bonds based on comparative analysis of the reduced and non-reduced peptide-sample. A: data derived for CysP2, B: data derived for CysP4.
(TIF)

File S1 Primers used for site directed mutagenesis.
(DOC)
