# Supplementary figures and images for: Stabilisation of the Fc Fragment of Human IgG1 by Engineered Intradomain Disulfide Bonds
Source: PLoS One. 2012 Jan 17;7(1):e30083. doi: 10.1371/journal.pone.0030083 (PMC3260182; doi:10.1371/journal.pone.0030083)

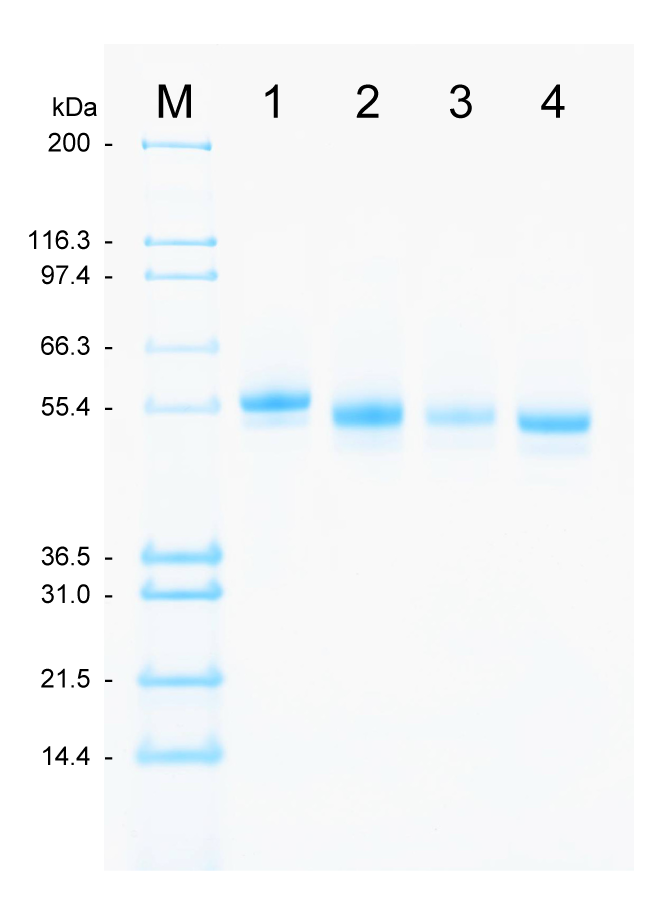

Supplement: Figure S1 — SDS-PAGE of purified wild-type Fc (lane 1), CysP4 (lane 2), CysP2 (lane 3) and CysP24 (lane 4). Molecular weight standard (lane M). (TIF) [file pone.0030083.s001.tif]

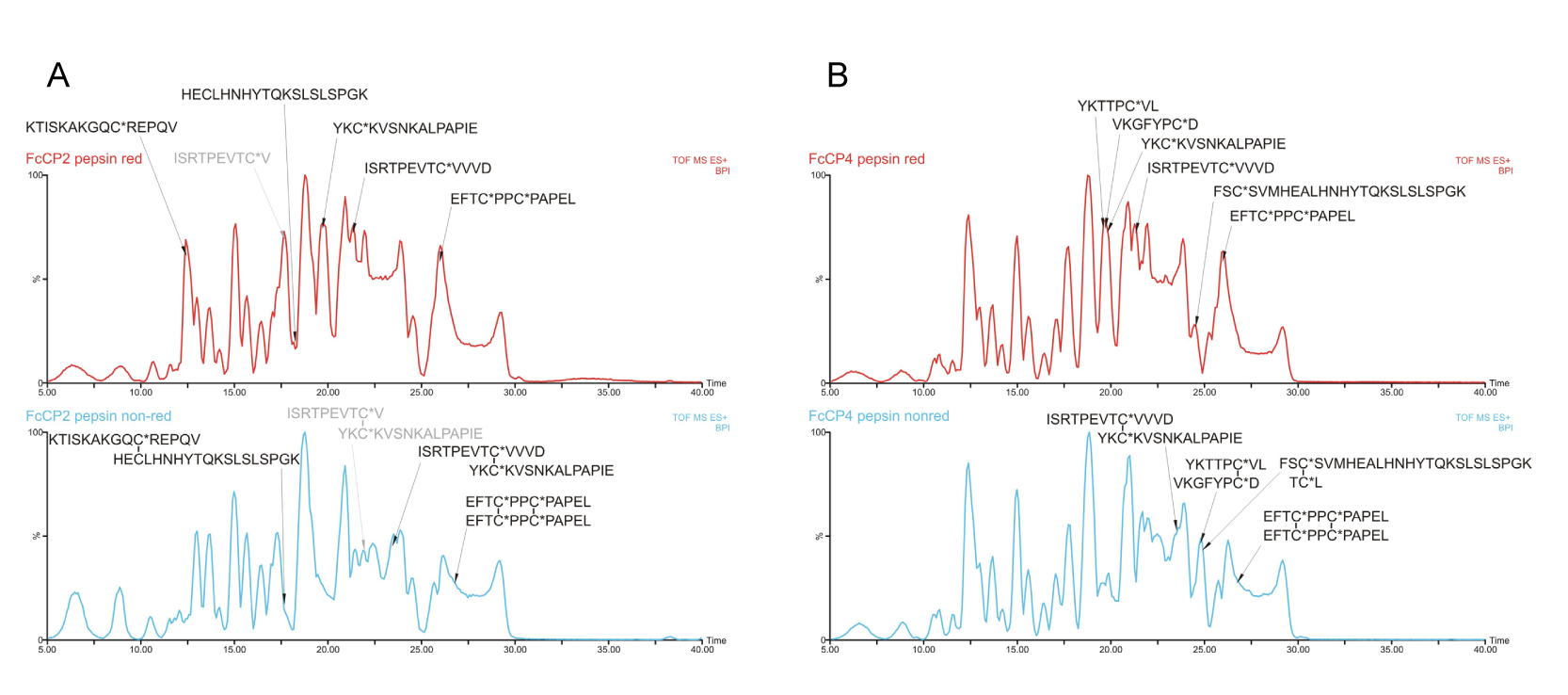

Supplement: Figure S2 — MS/MS data enabling prediction of disulfide bonds based on comparative analysis of the reduced and non-reduced peptide-sample. A: data derived for CysP2, B: data derived for CysP4. (TIF) [file pone.0030083.s002.tif]
