# Supplementary material for: Stabilisation of the Fc Fragment of Human IgG1 by Engineered Intradomain Disulfide Bonds
Source: PLoS One. 2012 Jan 17;7(1):e30083. doi: 10.1371/journal.pone.0030083 (PMC3260182; doi:10.1371/journal.pone.0030083)
Supplement: File S1 — Primers used for site directed mutagenesis. (DOC) [file pone.0030083.s003.doc]

## Primers

pP343C: 5-ctccaaagccaaagggcagtgtcgcgaaccacaggtgtacaccctgccc

pA431C: 5-gtcttctcatgctccgtgatgcacgagtgcctgcacaaccactacacacag

pS375C: 5-caagaaccaggtctcgttaacctgcctggtcaaaggcttctatccctgcgacatcgccgtggagtgggag

pP396C: 5-caactacaagaccacgccttgtgtgctggactccgacggatccttcttcctctacagcaagc

p136S375C: 5-ctctatggtgacgtctcgttaacctgcctggtcaaaggcttctatccctgcgacatcgccgtggagtgggag
